# Breast Cancer Presenting as Paraneoplastic Erythroderma: An Extremely Rare Case

**DOI:** 10.1155/2014/351065

**Published:** 2014-09-11

**Authors:** Ioannis Protopsaltis, Aspasia Drossou, Ioannis Katsantonis, Nikolaos Roussos, Kassiani Manoludaki, Miltiadis Arvanitis, Athanasia Papazafiropoulou, Stavros Antonopoulos

**Affiliations:** Department of Internal Medicine, Tzaneio General Hospital of Piraeus, 1 Zanni & Afentouli Street, 185 36 Piraeus, Greece

## Abstract

The skin may exhibit the first clinical evidence of a systemic disease and may provide the first clues to a diagnosis in malignancies. Erythroderma is defined as generalized redness and scaling and it is a clinical manifestation of a variety of underlying diseases including, rarely, solid tumors. Breast cancer is associated with a variety of skin paraneoplastic manifestations like acanthosis nigricans, erythromelalgia, thrombotic thrombocytopenic purpura, acrokeratosis paraneoplastica, dermatomyositis, systemic sclerosis, and scleroderma. However, in the literature, the correlation of erythroderma with breast cancer is quite infrequent. Here, we describe a case of a 76-year-old woman who presented with a paraneoplastic manifestation of erythroderma due to breast cancer.

## 1. Introduction

The skin may exhibit the first clinical evidence of a systemic disease and may provide the first clues to a diagnosis in up to 1% of internal malignancies [1]. Erythroderma, also known as the red man syndrome or exfoliative dermatitis, is defined as generalized redness and scaling which involves more than about 90% of body surface area. It is a clinical manifestation of a variety of underlying diseases including, rarely, solid tumors [2].

## 2. Case Presentation

**Figure 1:**
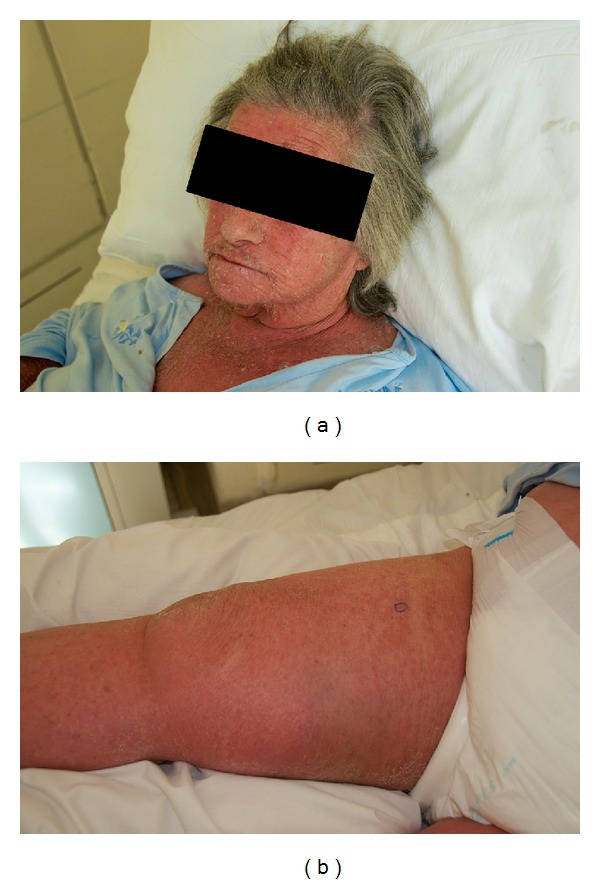
Symmetric generalized total body erythematous rash with prominent overlying scaling and desquamation involving the face (a) and leg (b).

A 76-year-old woman presented to our emergency department with worsening dyspnea and an insidious development of generalized cutaneous erythema (skin redness) (Figures 1(a) and 1(b)). There was no history of preexisting dermatoses or prior medical problems. Thorough questionnaire did not reveal any type of eczema or preexisting skin disease or any drug or herbal products, including any topical medication, which could have been overlooked by the patient.

Physical examination revealed symmetric generalized total body erythematous rash with prominent overlying scaling and desquamation involving the face, trunk, arms, and legs. Skin temperature was normal. No palpable lymph nodes were detected. Auscultation of the lungs revealed extended expiration and expiratory wheeze in the left hemithorax. Palpation of the left breast revealed a painless hard lump in the upper outer quadrant fixed to underlying structures. The results of the remainder of the examination were normal.

Laboratory tests were noteworthy for leukocytosis (34.94 k/*μ*L), thrombocytosis (550 k/*μ*L), anemia (Hct: 34.9%), and eosinophilia (3%). C-reactive protein was increased (27.4 mg/lt) as well as CA15.3 (97.3 U/mL). Chest radiograph revealed multiple bilateral nodular opacities, a finding that was confirmed by chest computed tomography, which showed multiple pulmonary nodules compatible with secondary malignant lesions. Additional finding was a lump sized approximately 8.5 cm in her left breast. All other laboratory tests were normal including urea, electrolytes, and serum IgE. According to the TNM Classification of Malignant Tumours, the patient was in stage T1N1M1.

**Figure 2:**
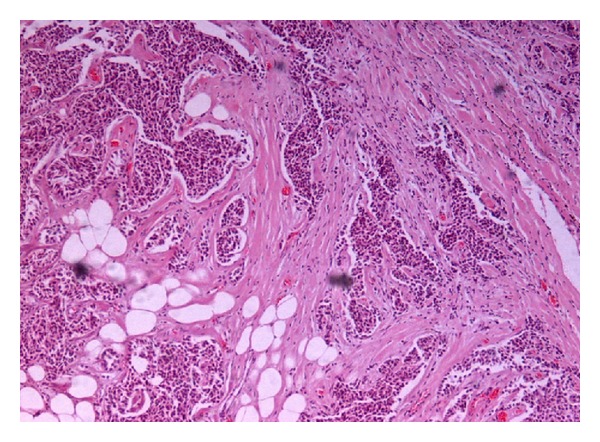
Breast biopsy (HE ×10). Invasive ductal carcinoma grade II.

**Figure 3:**
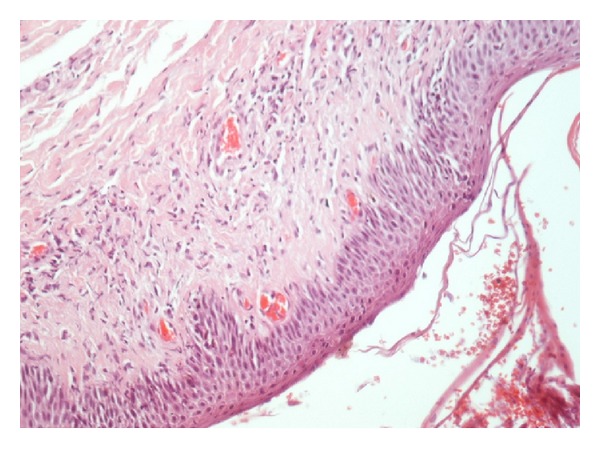
Skin biopsy. Extensive hyperemic vessels, a few extravasated erythrocytes, sparse inflammatory cells, and epidermis with mild parakeratosis and spongiosis.

Skin and breast biopsies were performed. Biopsy specimens of the skin revealed parakeratosis, hyperkeratosis, spongiosis, and scabbing of the epidermis while in upper dermis there was perivascular inflammatory infiltration mainly by eosinophils. No rete ridges elongation was prominent, strongly suggestive of the disease's acute phase. Biopsy of the mass in the left breast confirmed invasive ductal carcinoma NOS (not otherwise specified) grade 2 (immunohistochemistry was positive for hormone receptor of estrogens and ambiguous for HER-2/neu) (Figures 2 and 3).

The patient was treated with bronchodilators and fluid resuscitation; mometasone furoate emollient was applied for the cutaneous inflammation. However, following the previous treatment regimen, no rash amelioration was achieved, and the patient was started on high dose intravenous corticosteroids (prednisolone 1 mg/kg/day) for five days with distinct improvement. Finally, she was referred to oncology department for further management.

## 3. Discussion

Based on the above observations, a diagnosis of erythroderma as paraneoplastic manifestation secondary to breast cancer was made. To the best of our knowledge no previous reports have established this association after taking into account not only history and other clinical features but also confirmatory biopsy specimens of involved sites, taken from breast and skin.

The paraneoplastic dermatoses are relatively uncommon. Von Hebra first described generalized cutaneous inflammation in 1868 [3].The incidence of erythroderma is high in the Indian subcontinent although epidemiological data on a global scale are lacking. It presents more frequently between the ages of 61 and 70. It is more commonly seen in male patients, with a male to female ratio ranging from 2/1 to 4/1 [4, 5].

Erythroderma is a disorder which is commonly associated with preexisting dermatoses (psoriasis, atopic dermatitis, and contact dermatitis), drug-induced reactions, and occasionally internal malignancies [6]. Other causes of erythroderma include sarcoidosis, dermatomyositis, and hypereosinophilic syndrome while 25% of cases are considered idiopathic [7, 8]. Our patient reported no history of skin disease while she did not receive any medications that could be implicated as a likely erythroderma causative factor.

Malignancies constitute 1% of known causative factors. The most common of them are laryngeal, esophageal, or gastric, or those involving thyroid, lung, gallbladder, colon, fallopian tube, prostate, and lymphomas. There is also a distinct association with cutaneous T-cell lymphoma which comprises Sézary syndrome and mycosis fungoides [2, 9]. It should be mentioned that the correlation of erythroderma with underlying solid tumors is quite infrequent, and regarding breast cancer, to the best of our knowledge, only one case report has been published, lacking in not only tumor specific subtype report but also affected organs (skin breast) biopsy photos [10].

Breast cancer is associated with paraneoplastic manifestations from many systems. The most common skin paraneoplastic manifestations are acanthosis nigricans, erythromelalgia, thrombotic thrombocytopenic purpura, acrokeratosis paraneoplastica, paraneoplastic hypertrichosis lanuginosa acquisita, dermatomyositis, systemic sclerosis, and scleroderma [11–13].

In most tumor associated skin manifestations including erythroderma, there is no presence of neoplastic cells in the skin. Paraneoplastic exanthems might rather be attributed to a complicated interaction of cytokines (interleukin 1-2-8) and cellular adhesion molecules (VCAM-1, ICAM-1, E-selectin, and P-selectin), causing binding, transmigration, and infiltration of lymphocytes and mononuclear cells, which finally results in significant epidermal turnover rate [2, 14, 15]. Breast cancer is also a well-known source of such cytokine and cellular adhesion molecules overproduction [16, 17].

Our patient's clinical image was typical for erythroderma. She appeared with erythematous plaques witch eventually spread in most of her body. The patient's skin was bright red, dry, scaly, and warm to the touch, while she reported itching and skin tightness. Laboratory and histological findings, although nonspecific, were in line with those of erythroderma, such as mild anemia, leukocytosis with eosinophilia, high levels of CRP, and hypoalbuminemia [6, 18]. Skin biopsy findings were compatible with the usual histological ones of acute erythroderma (spongiosis parakeratosis, hyperkeratosis, acanthosis, and chronic perivascular inflammatory infiltrates).

Histopathology has been shown to help identify the cause in up to 50% of erythroderma cases. In particular lymphoma; hereditary disorders (ichthyosis, pityriasis rubra pilaris); various types of eczema, pemphigus, lichen planus, dermatophytosis, scabies, and dermatomyositis; and autoimmune diseases in general usually offer characteristic histopathologic findings. In the present case since microscopic findings remained nonspecific the paraneoplastic cause could reasonably be suspected.

Treatment of erythroderma is primarily supportive targeting to hydration and correction of electrolyte disturbances. Any inciting factor should be removed. Low dose topical steroids plus oral antihistamines are adequate in most cases, but not always [2, 9]. The course of the disease depends on the causative factor. In cases of drug-induced erythroderma, patients recover completely after drug discontinuation, while in the case of preexisting dermatoses, there is a slower course. During malignancies the removal of the tumor is imperative in order to achieve regression of skin manifestations [9].

In conclusion, erythroderma is a complex multifactorial dermatosis, the prognosis of which depends on the causative agent. Despite the fact that it appears rarely as a paraneoplastic syndrome, a clinical finding of a rapidly extending erythema along with various degrees of scaling, especially in a patient without any previous dermatological disorder, would warrant further investigation for underlying malignancies, as in this extremely rare case of breast cancer associated erythroderma.
